# O.4.6-6 Providing physical activity and sport opportunities in Active Schools: coordinators’ satisfaction with facilities and equipment

**DOI:** 10.1093/eurpub/ckad133.225

**Published:** 2023-09-11

**Authors:** Alexandra Valencia-Peris, Carmen Peiró-Velert, Carlos Evangelio, Javier Valenciano-Valcárcel, Esther Pérez-Gimeno

**Affiliations:** University of Valencia, Spain; University of Valencia, Spain; University of Valencia, Spain; University of Castilla La Mancha, Spain; carlos.evangelio@ext.uv.es; University of Castilla La Mancha, Spain; carlos.evangelio@ext.uv.es; University of Valencia, Spain

## Abstract

**Purpose:**

The premises and equipment available in schools are essential for developing programs that promote active lifestyles in schoolchildren. The aim of this study was to determine how satisfied coordinators of Active school programs were with their sports equipment and facilities and identify differences by type of school.

**Methods:**

In the 2021/22 academic year, 580 schools were accredited as Active schools in the Valencian Community (Spain). A representative sample of 257 Active school coordinators (33.1% women; 90.7% Physical Education teachers; 83.7% state schools) participated. An ad-hoc questionnaire was designed and administered on-line. It consisted of 47 open and closed response questions concerning the actions carried out to promote physical activity and sport and the school coordinators’ degree of satisfaction. The anonymity of all the participants was guaranteed. Descriptive statistics and chi-square tests were conducted on SPSS Statistics v. 26.0 software.

**Results:**

Active schools in the Valencian Community were equipped with the following facilities/spaces: sports fields or courts (55.6%), gym (55.5%), outdoor playing fields (41.4%), green spaces (10.9%), a football pitch (8.2%), skating rink (5.2%) and swimming-pool (0.8%). The Active school coordinators were quite satisfied with the school areas and playgrounds (65.3%) and sport equipment (75.1%) but dissatisfied with the gym or sports hall (60.7%), toilets and showers (59.5%), or with the equipment for students with functional diversity (66.1%). Significant differences by type of school emerged for school spaces and playgrounds (*χ*^*2*^_*(3)*_=11.480; *p*<.05), gym and sports hall (*χ*^*2*^_*(3)*_=10.829; *p*<.05) and equipment for students with functional diversity (*χ*^*2*^_*(3)*_=33.368; *p*<.05). Private schools were better represented in the maximum degree of satisfaction than state schools. The Active school coordinators suggested that improvements in outdoor premises (80.9%) and more and better equipment (74.7%) would contribute to increasing pupils' physical activity at school.

**Conclusions:**

The degree of satisfaction of the Active schools coordinators was balanced regarding the school equipment and facilities, but lower in state schools than private schools. The authorities should provide state schools with more sports and open spaces, green areas and appropriate equipment to facilitate designing inclusive, effective and sustainable physical activity and sports promotion programs.

**Funding:**

Grants AICO/2021/342 and Margarita Salas [MS2021].

